# Genetic variants of lncRNA HOTAIR and risk of epithelial ovarian cancer among Chinese women

**DOI:** 10.18632/oncotarget.8535

**Published:** 2016-04-28

**Authors:** Haijing Wu, Xiaofei Shang, Yu Shi, Zhirong Yang, Jun Zhao, Min Yang, Yan Li, Shiqiang Xu

**Affiliations:** ^1^ Department of Gynecologic Oncology, Sichuan Cancer Hospital, Chengdu, Sichuan, People's Republic of China; ^2^ The First Affiliated Hospital, Nanchang University, Nanchang, People's Republic of China

**Keywords:** long noncoding RNA, HOTAIR, genetic variants, ovarian cancer

## Abstract

Ovarian cancer is one of the common female malignant tumors globally. However, exactly mechanism of ovarian cancer remained unknown. HOTAIR, a lncRNA in the mammalian HOXC locus, has been fully explored for its genetic variants, expression level and carcinogenesis, development and progression of multiple cancers, except for ovarian cancer. In this study, we hypothesized that abnormal expression of HOTAIR and common variants of HOTAIR are associated with risk of Epithelial ovarian cancer (EOC). We first evaluated the HOTAIR levels in 100 paired tissues of EOC patients and corresponding normal tissues. Results showed that the expression level of HOTAIR in EOC tissues was significantly higher than that in corresponding normal tissues. Then the genotyping analyses of HOTAIR gene was conducted in a Chinese population. The results indicated that rs4759314 and rs7958904 were significantly associated with EOC susceptibility. For rs4759314, the difference between the G allele (as the reference) and the A allele was statistically significant (adjusted OR, 1.34; 95% CI: 1.08–1.65; *P* = 6.8 × 10^−3^). For rs7958904, C allele was associated a significantly decreased EOC risk when compared with G allele (OR: 0.77; 95% CI: 0.67–0.89; *P* = 4.2 × 10^−4^). The study identified that HOTAIR variants could be a useful biomarker for the predisposition to EOC and for the early diagnosis of the disease.

## INTRODUCTION

Ovarian cancer is the most lethal gynecologic malignancy and the fifth cause of cancer-related deaths among females worldwide [1]. It's reported that there will be totally 21,290 new cases and 14,180 new deaths in United States in 2015 [1]. Epithelial ovarian cancer (EOC) accounts for 90% to 95% of all cases of ovarian cancer [2, 3]. However, the etiology of EOC is not well understood but is likely to involve both genetic and environmental factors [4]. Therefore, discovery of new genes related to EOC risk and survival, as well as understanding their mechanism may provide important clues for, early detection, precise diagnosis and personalized therapy for EOC patients.

Recently, long non-coding RNAs (lncRNAs) have been focused for their wide range of biological regulatory functions. HOTAIR, a lncRNA located in the HOXC locus, has been fully explored for its genetic variants, expression level and carcinogenesis, development and progression of multiple cancers [5–12]. However, The specific role of HOTAIR in EOC susceptibility still remain unknown. Given the genetic variants and expression level of HOTAIR in carcinogenesis, development and progression of multiple cancers [13, 14], we hypothesized that common variants of HOTAIR are associated with risk of EOC. We therefore performed genotyping analyses of HOTAIR gene in this study conducted in Chinese population.

## RESULTS

### Characteristics of study subjects

The Clinical characteristics of the 1,000 patients with EOC and 1,000 controls are presented in Table 1. The average age of the EOC cases and the healthy controls was 65.2 and 64.8 years, respectively. No significant differences between the controls and cases were detected for age, drinking status or BMI. While significant differences were detected for smoking status and family history of cancer with *P* values smaller than 0.01.

**Table 1:** Clinical characteristics of the controls and patients

Variables	Patients (*n* = 1,000)	Controls (*n* = 1,000)	*P*-value
Age at diagnosis	65.2 ± 6.1	64.8 ± 4.7	0.100
Family history of cancer			
Yes	99	21	***P* < 0.001**
No	901	979	
Smoking status			
Never	877	918	**0.003**
Ever	123	82	
Drinking status			
Never	746	793	0.766
Ever	254	207	
Body mass index			
< 25 kg/m^2^	500	511	0.623
≥ 25 kg/m^2^	500	489	

### Associations of tagSNPs and EOC risk

The genotype distribution of all three tagSNPs (rs4759314, rs7958904 and rs874945) and their associations with EOC risk are shown in Table 2. The genotype distributions in the three SNPs were consistent with those expected from HWE among healthy controls (*P* > 0.05). For rs4759314, the carriers with the genotype AG have a 1.16-fold (95% CI: 0.90–1.51) risk of EOC and for those with the genotype AA have a 1.85-fold (95% CI: 1.11–3.09) risk of EOC, when compared to those with the genotype GG. When analyzed using the additive model, the trend was also significant (adjusted OR, 1.34; 95% CI: 1.08–1.65; *P* = 6.8 × 10^−3^). For rs7958904, carriers of C allele have a significantly decreased EOC risk when compared with those of G allele (OR: 0.77; 95% CI: 0.67 – 0.89; *P* = 4.2 × 10^−4^). The adjusted OR for the carriers of genotype CG was 0.84 (95% CI: 0.70–1.01) and 0.53 (95% CI: 0.37 – 0.75) for those with the genotype CC, when compared with those of genotype GG. We didn't detected any significant associations for rs874945. The stratified analyses by smoking status, drinking status and BMI were also conducted for rs4759314 and rs7958904 (Table 3). The trend didn't change materially.

**Table 2:** Genetic variants of HOTAIR and EOC risk

Genotype	Cases	Controls	Adjusted OR (95% CI)*
rs4759314			
AA	819	852	1.00 (reference)
AG	140	125	1.16 (0.90–1.51)
GG	41	23	1.85 (1.11–3.09)
G vs A			1.34 (1.08–1.65)
P trend			**6.8 × 10^−3^**
rs7958904			
GG	594	533	1.00 (reference)
CG	355	380	0.84 (0.70–1.01)
CC	51	87	0.53 (0.37–0.75)
C vs G			0.77 (0.67–0.89)
P trend			**4.2 × 10^−4^**
rs874945			
GG	665	677	1.00 (reference)
AG	283	279	1.03 (0.85–1.26)
AA	52	44	1.20 (0.79–1.82)
A vs G			1.07 (0.91–1.25)
P trend			0.382

* Asjusting for age at diagnosis, family history of cancer, smoking status, drinking status, and BMI.

**Table 3:** Genetic variants of HOTAIR and EOC risk stratified by co-variables

Variables	rs4759314 (G vs A)	rs7958904 (C vs G)
Body mass index		
≥ 25 kg/m^2^	1.33 (1.07–1.65)	0.76 (0.63–0.91)
P trend	**0.010**	**0.003**
< 25 kg/m^2^	1.34 (1.07–1.67)	0.79 (0.67–0.93)
P trend	**0.009**	**0.005**
**Smoking status**		
Smokers	1.35 (1.08–1.68)	0.76 (0.65–0.89)
P trend	**0.008**	**8.2 × 10^−4^**
Non-smokers	1.33 (0.85–2.07)	0.78 (0.46–1.33)
P trend	0.211	0.361
**Drinking status**		
Drinkers	1.33 (1.07–1.65)	0.76 (0.63–0.94)
P trend	**0.010**	**0.009**
Non-drinkers	1.36 (0.86–2.15)	0.79 (0.54–1.15)
P trend	0.189	0.223

### Quantitative real-time RT-PCR analyses of HOTAIR

**Figure 1:**
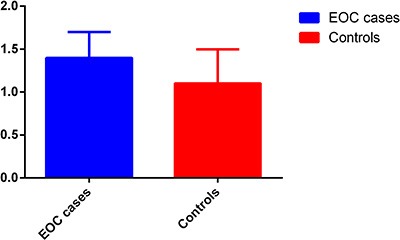
Quantitative real-time RT-PCR analyses of HOTAIR

To confirm the functional relevance and abnormal expression of HOTAIR in EOC patients, we first evaluated the HOTAIR levels in 100 paired tissues of EOC patients and paired normal tissues. The expression level of lncRNA HOTAIR in EOC tissues was significantly higher than that in paired normal tissues (Figure 1, *P* < 0.001).

## DISCUSSION

In current study, we first evaluated the HOTAIR levels in paired tissues of EOC patients and corresponding normal tissues. The expression level of lncRNA HOTAIR in EOC tissues was significantly higher than that in paired normal tissues. We then evaluated three tagSNPs SNPs of HOTAIR gene to investigate whether these SNPs are related to EOC risk in a Chinese Han population. Our results demonstrate that rs4759314 and rs7958904 were significantly associated with EOC susceptibility. To our knowledge, this should be the first study which aims to examine the association between genetic variants of lncRNA HOTAIR and EOC risk in a relatively large group of Asians.

LncRNAs are involved in many human disease, and could drive the development and progression of the diseases [8, 15–23]. The lncRNA HOTAIR could modify the progress of carcinogenesis [7, 12, 20]. HOTAIR (for HOX transcript antisense RNA), was located on chromosome 12: 53.96–53.97 Mb [15]. The gene length of lncRNA HOTAIR gene was 6,232 bp. Loss of HOTAIR could restrain invasiveness of cancers [12]. Finally, through these approach, HOTAIR gene could regulate the progress of carcinogenesis and its development.

In current study, we detected significant associations between the rs4759314 and rs7958904 and EOC susceptibility among Chinese population, some studies have also demonstrated significant associations between rs7958904 and decreased risk of colorectal cancer [7], rs4759314 with the increased gastric cancer risk [20]. The major strength of this study is the use of a large sample size to study genetic predispositions, which are less likely to be affected by confounding factors. Another strength is our sufficient demographic information. In summary, the present study showed significant associations between HOTAIR rs4759314 and rs7958904 and the risk of EOC.

## MATERIALS AND METHODS

### Subjects

Included in this study were 1,000 continuous subjects diagnosed with EOC through a rapid case-ascertainment system that were diagnosed from 2010 to 2015, and 1,000 controls were identified and frequency matched by 5-year age groups and resident regions. A histopathological diagnosis was made by an experienced pathologist. Blood samples (10 ml) were obtained from the subjects who participated in the study. Clinical information of patients were collected from medical records, and a structured questionnaire was used to elicit detailed information on demographic factors. All specimens were handled and made anonymous according to the ethical and legal standards. Approval was granted from relevant review boards, and informed consent was granted by all included participants.

### SNP selection and genotyping

Using data of HapMap Chinese Han Beijing (CHB), we selected the tagSNPs capturing all the common SNPs (minor allele frequency, MAF > 0.05) located in the chromosome locus transcribed into HOTAIR and its flanking 2000bp region with *r*^2^ > 0.8. Finally, three SNPs were selected for genotyping (rs4759314, rs7958904 and rs874945). Genomic DNA was extracted from peripheral blood leukocytes using the GoldMag^®^ nanoparticles method (GoldMag Ltd. Xi'an, China) according to the manufacturer's instructions, and DNA concentration was measured using the NanoDrop 2000 (Thermo Scientific, Waltham, Massachusetts, USA). Sequenom MassARRAY Assay Design 3.0 Software was used to design Multiplexed SNP MassEXTEND assays. SNP genotyping was performed by the Sequenom MassARRAY RS1000 while Sequenom Typer 4.0 Software was used to perform data management and analysis.

### Quantitative real-time RT-PCR analyses of HOTAIR

The expression of HOTAIR from 100 EOC tissues and adjacent normal tissues were determined by SYBR Green Assay and the levels were normalized by β-actin by the 2−ΔCt method. All assays were conducted by using the ABI 7900 HT real-time PCR system (Applied Biosystems, Foster City, CA, USA).

### Statistical analyses

Differences in the distribution of selected demographic variables and genotypes of tagSNPs were evaluated by Pearson's χ^2^ test. Hardy-Weinberg equilibrium (HWE) for each SNP among controls was tested using a goodness-of-fit χ^2^-test. The associations of each SNP and EOC susceptibility were estimated by using unconditional logistic regression analyses with odds ratios (ORs) and 95% confidence intervals (CIs). Statistical analysis was conducted using the SPSS 19.0 for Windows (SPSS Inc., Chicago, IL, USA). All *p* values presented in this study are two-sided, and we used *p* < 0.05 as the threshold of statistical significance.
